# Simulated Pediatric Blood Cultures to Assess the Inactivation of Clinically Relevant Antimicrobial Drug Concentrations in Resin-Containing Bottles

**DOI:** 10.3389/fcimb.2021.649769

**Published:** 2021-03-19

**Authors:** Liliana Giordano, Flora Marzia Liotti, Giulia Menchinelli, Giulia De Angelis, Tiziana D’Inzeo, Grazia Angela Morandotti, Maurizio Sanguinetti, Teresa Spanu, Brunella Posteraro

**Affiliations:** ^1^ Dipartimento di Scienze Biotecnologiche di Base, Cliniche Intensivologiche e Perioperatorie, Università Cattolica del Sacro Cuore, Rome, Italy; ^2^ Dipartimento di Scienze di Laboratorio e Infettivologiche, Fondazione Policlinico Universitario A. Gemelli IRCCS, Rome, Italy; ^3^ Dipartimento di Scienze Mediche e Chirurgiche, Fondazione Policlinico Universitario A. Gemelli IRCCS, Rome, Italy

**Keywords:** bacteremia, antimicrobial drugs, simulated blood cultures, pediatric patients, therapeutic drug concentrations

## Abstract

The bacteremia level as well as the administration of antibiotics before blood collection may significantly affect the recovery of bacterial pathogens from pediatric blood cultures in BacT/Alert Virtuo or Bactec FX BC systems, which remain the common techniques to diagnose bacteremia in pediatric patients. We simulated pediatric blood cultures with low or intermediate bacteremia level to evaluate BacT/Alert PF Plus and Bactec Peds Plus blood culture bottles for resin-based inactivation of 16 antibiotic–bacterium combinations. Overall, 105/192 (54.7%) of BacT/Alert PF Plus bottles and 69/192 (36.0%) of Bactec Peds Plus bottles allowed organisms to grow when exposed to antibiotics. In particular, both BacT/Alert PF Plus and Bactec Peds Plus bottles proved to be effective with piperacillin/tazobactam and *Pseudomonas aeruginosa* or with oxacillin and methicillin-susceptible *Staphylococcus aureus* (100% growth), whereas no effectiveness was apparent with ceftriaxone and *Escherichia coli*, *Streptococcus agalactiae*, or *Streptococcus pneumoniae* or with cefepime and *E. coli* (0% growth). In some relevant instances (*e.g.*, with vancomycin and methicillin-resistant *S. aureus* or *Streptococcus pneumoniae*), BacT/Alert PF Plus bottles were superior to Bactec Peds Plus bottles. Together, these findings underscore the potentiality of resin-containing bottles to enhance diagnosis of bacteremia in pediatric patients on antimicrobial therapy. This is particularly true with one of the evaluated BC systems and with simulated intermediate bacteremia level only.

## Introduction

Bloodstream infection (BSI) remains a major cause of morbidity and mortality in pediatric patients (Weiss et al., 2015), especially in neonates (Shane et al., 2017) and hematology/oncology patients (Dandoy et al., 2019). Like in adult populations, blood culture (BC) coupled with continuous monitoring in currently available BC systems (BacT/Alert Virtuo [bioMérieux, Marcy l’Étoile, France] and Bactec FX [Becton Dickinson, Sparks, MD]) is the common technique to diagnose bacteremia in either infants or children (Wilson, 2020a). Besides being a prerequisite for targeted antibiotic administration (Liu et al., 2017), BC may allow for cessation of unnecessary empirical antimicrobial therapy in case of negative results (Dien Bard and McElvania TeKippe, 2016). However, low-level [*i.e.*, ≤10 CFU per milliliter (CFU/ml) of blood] bacteremia (Kellogg et al., 2000) as well as inadequate blood volume sampling (based on body weight) (Huber et al., 2020) may significantly compromise the recovery of BSI pathogens (including fastidious organisms) from pediatric BC bottles (Dien Bard and McElvania TeKippe, 2016). While inadequate sampling may be overcome by strictly adhering to BC system manufacturer’s recommendations, importantly, antibiotic administration before obtaining BCs frequently occurs (Cecconi et al., 2018) and may lower the yield of positive results (Zadroga et al., 2013).

A technological advance to optimize BC performance (Wilson, 2020b) is the introduction of specialized “Plus” BC bottles that contain antibiotic binding resins (Flayhart et al., 2007; Roh et al., 2012; Chung et al., 2019; Menchinelli et al., 2019a; Menchinelli et al., 2019b; Xu et al., 2021). In previous formulations, pediatric BC bottles marketed by bioMérieux—namely BacT/Alert Pediatric FAN (PF) BC bottles—contained 8.5% charcoal suspension as an antibiotic-neutralizing (or inactivating) substance. The only recently published bacteremia simulating study in the pediatric setting (Dien Bard and McElvania TeKippe, 2016) has compared BacT/Alert PF (bioMérieux) and Bactec Peds Plus (Becton Dickinson) BC bottles for their efficacy against eight organism-appropriate antibiotics (Sullivan et al., 2013). Consistently, studies investigating the effect of antibiotic-inactivating substances using the newer BacT/Alert PF Plus BC bottle are lacking.

The aim of this study was to evaluate both BacT/Alert PF Plus and Bactec Peds Plus bottles for the *in vitro* inactivation of clinically relevant antimicrobial drug concentrations using a pediatric BC simulation model. We tested different antibiotic–organism combinations in BC bottles inoculated with 2 ml human blood, which was consistent with a BC system manufacturers’ validated 1–4 ml optimum to detect BSI pathogens (Huber et al., 2020).

Part of these results would have been presented as an Abstract at the 30th European Congress of Clinical Microbiology and Infectious Diseases that would have taken place in Paris, France, 2020, but which was cancelled.

## Materials and Methods

### Study Design, Organisms, and Simulated Cultures

In this BC simulation study, eight aerobic or facultative anaerobic organisms (*Escherichia coli* ATCC^®^ 25922™, *Pseudomonas aeruginosa* ATCC^®^ 27853™, methicillin-resistant *Staphylococcus aureus* [MRSA] ATCC^®^ 43300™, methicillin-susceptible *Staphylococcus aureus* [MSSA] ATCC^®^ 29213™, *Staphylococcus capitis* ATCC^®^ 146™, *Staphylococcus epidermidis* ATCC^®^ 12228™, *Streptococcus agalactiae* ATCC^®^ 12386™, and *Streptococcus pneumoniae* ATCC^®^ 49619™) were used. Bacteria were suspended in phosphate-buffered saline to achieve a McFarland density of 0.5 (equivalent to ~1 × 10^8^ CFU/ml). After three successive 100-fold dilutions, two aliquots of bacterial suspension, one containing ~10 CFU/ml and the other one containing ~60 CFU/ml, were prepared. Then, 0.5 ml from each aliquot was inoculated into BacT/Alert PF Plus or Bactec Peds Plus bottle with 2 ml of banked human whole blood (obtained from the Division of Transfusion Medicine of our institution). This allowed simulating, for each series of bottles, bacteremia levels of 2–3 CFU (low) or 14–16 CFU (intermediate) per ml of blood, respectively. We defined as low or intermediate those levels that, albeit at a different extent, were considerably below the 50 CFU per ml of blood reported to be high level in children (Kellogg et al., 2000). Despite being arbitrary, this categorization was reflective of bacteremia levels frequently encountered in pediatric patients (Dien Bard and McElvania TeKippe, 2016). Because the volume of growth medium was 30 ml in the BacT/Alert PF Plus bottle and 40 ml in the Bactec Peds Plus bottle, final CFU numbers per bottle dropped proportionally. In each experiment day, the inoculum size was confirmed by plating each organism’s suspension on standard solid media. Based on relevance (Amsden, 2010; Sullivan et al., 2013), we chose eight antimicrobial drugs (ampicillin, cefepime, ceftriaxone, gentamicin, meropenem, oxacillin, piperacillin-tazobactam, and vancomycin) as representative of commonly used antibiotics for the empirical antimicrobial therapy of suspected BSIs at our institution’s pediatrics clinic. We purchased ampicillin, ceftriaxone, and vancomycin from Sigma-Aldrich (Saint Louis, MO, USA), cefepime, meropenem, and oxacillin from U.S. Pharmacopeia (Rockville, MD, USA), gentamicin and tazobactam from TOKU-E (Bellingham, WA, USA), and piperacillin from MP Biomedicals (Solon, OH, USA). Based on their MICs (**Table 1**, footnote b), all bacterial organisms displayed full susceptibility to the drugs specifically tested against them (ISO, 2006; CLSI, 2020). For each antimicrobial drug, a four-fold stock solution was prepared and diluted to reach final (peak or trough) concentrations (**Table 1**). For each antibiotic, concentrations were equal to the estimated therapeutic levels (Amsden, 2010; Sullivan et al., 2013) and were above the corresponding MIC value determined for each bacterial organism (**Table 1**, footnote b). Finally, BacT/Alert PF Plus or Bactec Peds Plus bottles were inoculated with 2 ml of blood, followed by 0.5 ml of the antibiotic suspension and 0.5 ml of the organism suspension (both prepared as described above). Bottles containing antibiotic-free whole blood were or not inoculated with bacterial organisms and were included as controls.

**Table 1:** Detection results of organisms in resin-containing BacT/Alert or Bactec pediatric blood culture bottles according to antimicrobial drug and relative concentration.^a^

Organisms in the presence of indicated drugs	Peak (P) or trough (T) drug concentration (µg/ml)^b^	Recovery by no. replicates/total (mean TTD [h]) for:		ΔTTD (h) for:		Recovery by no. replicates/total (mean TTD [h]) for:		ΔTTD (h) for:	
		BacT/Alert PF Plus bottles at a simulated bacteremia level of:				Bactec Peds Plus bottles at a simulated bacteremia level of:			
		2–3 CFU/ml	14–16 CFU/ml	2–3 CFU/ml	14–16 CFU/ml	2–3 CFU/ml	14–16 CFU/ml	2–3 CFU/ml	14–16 CFU/ml
*Escherichia coli*									
Cefepime	P (164)	0/3	0/3	NA	NA	0/3	0/3	NA	NA
	T (10)	0/3	0/3	NA	NA	0/3	0/3	NA	NA
	No drug	3/3 (10.90)	3/3 (9.35)			3/3 (11.97)	3/3 (10.72)		
Ceftriaxone	P (250)	0/3	0/3	NA	NA	0/3	0/3	NA	NA
	T (94)	0/3	0/3	NA	NA	0/3	0/3	NA	NA
	No drug	3/3 (10.03)	3/3 (10.01)			3/3 (11.97)	3/3 (10.42)		
Meropenem	P (58.5)	0/3	0/3	NA	NA	0/3	0/3	NA	NA
	T (0.2)	0/3	3/3 (14.49)	NA	3.78	0/3	3/3 (19.43)	NA	7.75
	No drug	3/3 (12.91)	3/3 (10.71)			3/3 (15.62)	3/3 (11.68)		
*Pseudomonas aeruginosa*									
Cefepime	P (164)	0/3	3/3 (26.25)	NA	11.79	0/3	0/3	NA	NA
	T (10)	3/3 (15.31)	3/3 (15.05)	0.26	0.59	3/3 (17.40)	3/3 (15.08)	0.20	0.00
	No drug	3/3 (15.05)	3/3 (14.46)			3/3 (17.20)	3/3 (15.08)		
Gentamicin	P (8)	0/3	3/3 (15.05)	NA	0.59	0/3	3/3 (15.58)	NA	0.30
	T (1)	3/3 (15.31)	3/3 (15.12)	0.18	0.66	3/3 (17.4)	3/3 (15.08)	0.28	−0.20
	No drug	3/3 (15.33)	3/3 (14.46)			3/3 (17.12)	3/3 (15.28)		
Meropenem	P (58.5)	0/3	0/3	NA	NA	0/3	0/3	NA	NA
	T (0.2)	0/3	3/3 (13.22)	NA	0.11	0/3	3/3 (17.40)	NA	3.34
	No drug	3/3 (12.12)	3/3 (13.11)			3/3 (17.34)	3/3 (14.06)		
Piperacillin-tazobactam	P (240/24)	3/3 (16.11)	3/3 (14.49)	1.99	1.38	3/3 (17.58)	3/3 (16.10)	1.24	1.04
	T (5/0.7)	3/3 (15.53)	3/3 (14.12)	1.41	1.01	3/3 (17.58)	3/3 (16.22)	1.24	1.16
	No drug	3/3 (14.12)	3/3 (13.11)			3/3 (16.34)	3/3 (15.06)		
Methicillin-resistant *Staphylococcus aureus*									
Vancomycin	P (50)	3/3 (15.00)	3/3 (13.54)	4.83	4.87	0/3	3/3 (16.54)	NA	6.32
	T (10)	3/3 (14.59)	3/3 (13.20)	4.42	4.53	3/3 (17.59)	3/3 (15.20)	6.40	4.98
	No drug	3/3 (10.17)	3/3 (8.67)			3/3 (11.19)	3/3 (10.22)		
Methicillin-susceptible *Staphylococcus aureus*									
Oxacillin	P (230)	3/3 (11.56)	3/3 (9.89)	0.30	0.00	3/3 (15.62)	3/3 (15.29)	0.08	4.39
	T (13)	3/3 (11.55)	3/3 (10.71)	0.29	0.82	3/3 (15.88)	3/3 (12.67)	0.34	1.77
	No drug	3/3 (11.26)	3/3 (9.89)			3/3 (15.54)	3/3 (10.90)		
Vancomycin	P (50)	0/3	3/3 (15.07)	NA	3.94	0/3	3/3 (21.27)	NA	10.35
	T (10)	3/3 (16.22)	3/3 (13.43)	4.96	2.30	0/3	3/3 (17.02)	NA	3.02
	No drug	3/3 (11.26)	3/3 (11.13)			3/3 (15.54)	3/3 (10.92)		
*Staphylococcus capitis*									
Vancomycin	P (50)	0/3	3/3 (17.54)	NA	3.43	0/3	0/3	NA	NA
	T (10)	3/3 (18.35)	3/3 (16.59)	3.24	2.48	0/3	3/3 (17.59)	NA	2.06
	No drug	3/3 (15.11)	3/3 (14.11)			3/3 (16.12)	3/3 (15.53)		
*Staphylococcus epidermidis*									
Vancomycin	P (50)	0/3	3/3 (17.32)	NA	3.22	0/3	0/3	NA	NA
	T (10)	3/3 (18.23)	3/3 (16.39)	2.90	2.29	0/3	3/3 (17.55)	NA	2.02
	No drug	3/3 (15.33)	3/3 (14.10)			3/3 (16.12)	3/3 (15.53)		
*Streptococcus agalactiae*									
Ampicillin	P (47)	3/3 (12.13)	3/3 (11.20)	3.01	3.08	0/3	0/3	NA	NA
	T (3)	3/3 (10.47)	3/3 (10.38)	1.35	2.26	3/3 (13.49)	3/3 (12.33)	3.30	3.21
	No drug	3/3 (9.12)	3/3 (8.12)			3/3 (10.19)	3/3 (9.12)		
Ceftriaxone	P (250)	0/3	0/3	NA	NA	0/3	0/3	NA	NA
	T (94)	0/3	0/3	NA	NA	0/3	0/3	NA	NA
	No drug	3/3 (9.22)	3/3 (8.31)			3/3 (10.11)	3/3 (9.32)		
*Streptococcus pneumoniae*									
Ceftriaxone	P (250)	0/3	0/3	NA	NA	0/3	0/3	NA	NA
	T (94)	0/3	0/3	NA	NA	0/3	0/3	NA	NA
	No drug	3/3 (13.65)	3/3 (13.32)			3/3 (15.05)	3/3 (14.82)		
Vancomycin	P (50)	0/3	3/3 (15.65)	NA	4.17	0/3	0/3	NA	NA
	T (10)	0/3	3/3 (15.93)	NA	4.45	0/3	0/3	NA	NA
	No drug	3/3 (13.43)	3/3 (11.48)			3/3 (14.99)	3/3 (14.44)		

a Mean time to detection (TTD) was calculated on three replicates of simulated blood cultures for each of listed organism–antimicrobial drug combinations; ΔTTD was calculated by difference of the mean TTD of bottles with any antimicrobial drug concentration and the mean TTD of bottles (controls) without antimicrobial drug; NA, not applicable.

b For each antimicrobial drug tested, P or T concentrations were equal to the estimated therapeutic levels reported elsewhere (Amsden, 2010; Sullivan et al., 2013), and were above the MIC values for listed bacterial organisms as reported in the documents by EUCAST (http://www.eucast.org/fileadmin/src/media/PDFs/EUCAST_files/Breakpoint_tables/v_10.0_Breakpoint_Tables.pdf) or, only for ceftriaxone and S. agalactiae, CLSI (2020). For ampicillin, MIC value was 0.06 (S. agalactiae); for cefepime, MIC values were 0.06 µg/ml (E. coli) and 2 µg/ml (P. aeruginosa); for ceftriaxone, MIC values were 0.06 µg/ml (E. coli), 0.25 µg/ml (S. agalactiae), and 0.06 µg/ml (S. pneumoniae); for gentamicin, MIC value was 2 µg/ml (P. aeruginosa); for meropenem, MIC values were 0.06 µg/ml (E. coli) and 0.12 µg/ml (P. aeruginosa); for oxacillin, MIC value was 0.12 µg/ml (methicillin-susceptible S. aureus); for piperacillin-tazobactam, MIC value was 4/4 µg/ml (P. aeruginosa); and for vancomycin, MIC values were 1 µg/ml (methicillin-resistant S. aureus, methicillin-susceptible S. aureus, S. capitis, or S. epidermidis) and 0.25 µg/ml (S. pneumoniae), respectively.

In total, we tested in triplicate antibiotic–organism combinations with their respective controls at each of two bacteremia levels. Shortly after inoculation, BacT/Alert PF Plus or Bactec Peds Plus bottles were loaded into the respective BacT/Alert Virtuo or Bactec FX BC instruments, and then incubated for a maximum of 5 days (120 h) or until growth was detected. A positive signal (growth detection) within 120 h was used as the endpoint to calculate the time to detection (TTD) for each bottle (*i.e.*, the time from the bottle loading into the instrument to a positive signal). Bottles that remained negative at 120 h were recorded as “no growth”. Standard subcultures from any BC bottle were performed to confirm positive or negative detection results and/or to exclude possible contaminations.

### Data Analysis

Results were reported as percentages of recovered organisms or as mean TTDs in each BC system, and differences between results in predefined groups were assessed using the McNemar’s test or the paired *t* test, as appropriate. Statistical analyses were performed using the Intercooled Stata program version 11 and GraphPad Prism 7, and a *P* value of <0.05 was considered statistically significant.

## Results

**Figure 1:**
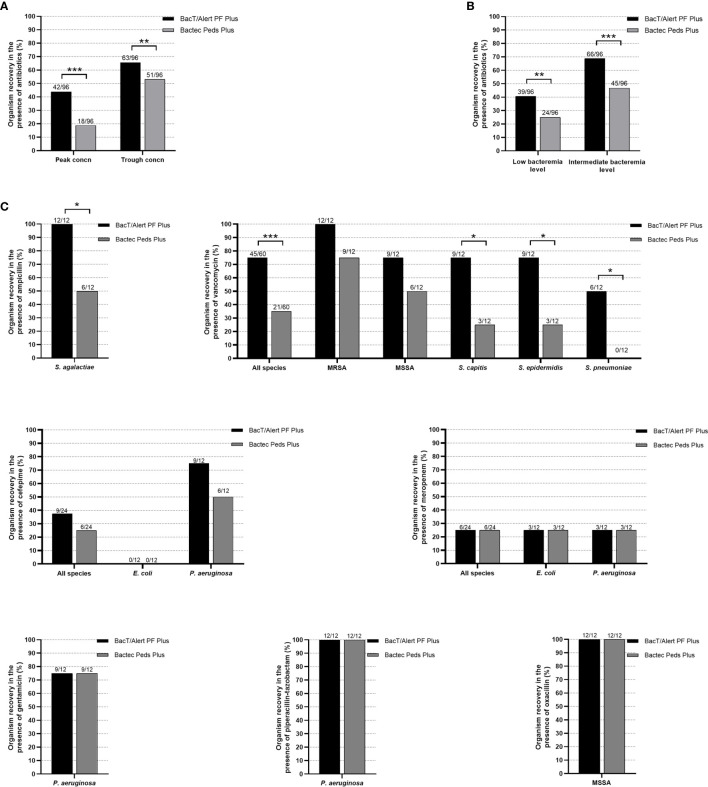
Percentages of organism recovery from BacT/Alert PF Plus or Bactec Peds Plus BC bottles inoculated with antibiotic-containing blood as stratified by antibiotics peak or trough concentrations **(A)**, low or intermediate bacteremia levels **(B)**, and antibiotic–organism combinations **(C)**, respectively. Percentages are relative to the organism recovery from the total of antibiotic-containing BC bottles inoculated for each indicated group. All (100%) of BC bottles without antibiotics used as controls yielded organism recovery (data not shown). Asterisks indicate the statistical significance of comparisons between BC bottles (^*^ *P* = 0.01; ^**^ *P <*0.001; ^***^ *P <*0.0001). Comparisons with *P* values of >0.05 were not indicated.

Among 576 simulated BacT/Alert PF Plus or Bactec Peds Plus BC bottles, 384 bottles were tested with bacterial organisms in the presence of clinically relevant peak (192 bottles) or trough (192 bottles) antibiotics’ concentrations, whereas 192 bottles (controls) were tested in the absence of antibiotics. As expected, 100% (192/192) of controls yielded all the organisms included in the study. Conversely, growth was detected in 105/192 (54.7%) of BacT/Alert PF Plus bottles and in 69/192 (36.0%) of Bactec Peds Plus bottles with organisms exposed to antibiotics. As shown in **Figure 1A**, percentages of organism recovery between bottles (BacT/Alert PF Plus versus Bactec Peds Plus) were significantly different when stratifying results by antimicrobial drug peak [43.8% (42/96) *versus* 18.8% (18/96), *P <*0.0001] or trough [65.6% (63/96) *versus* 53.1% (51/96), *P* = 0.0005) concentrations. Similarly (**Figure 1B**), significant differences were seen in the percentage of organism recovery when stratifying results by simulated bacteremia low [40.6% (39/96) *versus* 25.0% (24/96), *P* = 0.0001] or intermediate [68.8% (66/96) *versus* 46.9% (45/96), *P <*0.0001] levels. Excluding ceftriaxone—no organisms were recovered when exposed to this drug (**Table 1**)—recovery results in BacT/Alert PF Plus *versus* Bactec Peds Plus bottles were shown by thirteen antibiotic–organism combinations (**Figure 1C**). Thus, percentages of *S. agalactiae* recovery in the presence of ampicillin differed significantly [100% (12/12) *versus* 50.0% (6/12), *P* = 0.01] as well as were those for all species [75.0% (45/60) *versus* 35.0% (21/60), *P <*0.0001], *S. capitis* [75.0% (9/12) *versus* 25.0% (3/12), *P* = 0.01], *S. epidermidis* [75.0% (9/12) *versus* 25.0% (3/12), *P* = 0.01], or *S. pneumoniae* [50.0% (6/12) *versus* 0.0% (0/12), *P* = 0.01], respectively, exposed to vancomycin (**Figure 1C**).

As shown in **Table 1**, the BacT/Alert PF Plus bottles allowed organism recovery in drug concentrations that Bactec Peds bottles did not. Some bottles (simulating low-level bacteremia with the indicated organism) contained vancomycin peak (MRSA), vancomycin trough (MSSA, *S. capitis*, or *S. epidermidis*), or ampicillin peak (*S. agalactiae*) concentrations. The remaining bottles (simulating intermediate-level bacteremia with the indicated organism) contained cefepime peak (*P. aeruginosa*), ampicillin peak (*S. agalactiae*), vancomycin peak (*S. capitis*, *S. epidermidis*, or *S. pneumoniae*), or vancomycin trough (*S. pneumoniae*) concentrations. In both BacT/Alert PF Plus and Bactec Peds Plus bottles, drugs inhibited growth of *E. coli* (cefepime and ceftriaxone) and of *S. agalactiae* and *S. pneumoniae* (ceftriaxone), whereas meropenem inhibited growth of *E. coli* and *P. aeruginosa* only in low-level bacteremia simulating bottles. Of note, the two organisms were shown to overcome the meropenem inhibition in intermediate-level bacteremia simulating bottles. Conversely, both BacT/Alert PF Plus and Bactec Peds Plus bottles allowed *P. aeruginosa* and MSSA to grow in the presence of piperacillin/tazobactam or oxacillin, respectively. Overall, the mean TTD was 14.71 h (range, 9.89 to 26.25 h) with the BacT/Alert PF Plus bottle and 16.37 h (range, 12.33 to 21.27 h) with the Bactec Peds Plus bottle (*P* = 0.04). As expected, ΔTTDs ≥3 h between bottles with drug and the paired controls (no-drug bottles) were observed with either BacT/Alert PF Plus or Bactec Peds Plus bottles whereas, in some cases, both bottle types had extended ΔTTD. These included vancomycin peak (intermediate-level MRSA or MSSA bacteremia) and trough (low/intermediate-level MRSA bacteremia) concentrations or trough meropenem (intermediate-level *E. coli* bacteremia) concentration.

## Discussion

Our findings add to the information recently published on resin-containing bottles BacT/Alert Plus and Bactec Plus in simulated BCs. These so called “Plus” bottle formulations are able to support and/or enhance the growth of several pathogens commonly isolated from blood (Wilson, 2020b). In simulated low (2–3 CFU/ml) or intermediate (14–16 CFU/ml) bacteremia levels, BacT/Alert PF Plus or Bactec Peds Plus bottles recovered all bacterial species (*E. coli*, *P. aeruginosa*, four staphylococcal species, and two streptococcal species) in the absence of antimicrobial drugs (192 of 192 bottles). BacT/Alert PF Plus or Bactec Peds Plus bottles led to satisfactory organisms’ recovery rates when inoculated with bacterial species and antimicrobial drugs to which they were susceptible. This was because of resins’ binding kinetics that was capable to quickly lower drug concentrations to below organisms’ MICs.

To the best of our knowledge, this is the first study to evaluate BacT/Alert PF Plus bottles after 2013, the year these bottles became commercially available. Indeed, published studies until now (Chen et al., 2019; Chung et al., 2019; Menchinelli et al., 2019b; Xu et al., 2021) have simulated adult BCs—using human, horse, or sheep blood sources—to assess the antibiotic inactivation capability of Bactec Plus Aerobic/F or Anaerobic/F bottles in comparison with BacT/Alert FA Plus (aerobic) or FN Plus (anaerobic) bottles. In our study, both BacT/Alert PF Plus and Bactec Peds Plus bottles showed 100% recovery in two instances (piperacillin/tazobactam with *P. aeruginosa* and oxacillin with MSSA), which suggests that antibiotic-binding resins might be effective regardless of bacteria or antibiotic levels in blood samples. In other instances, both BacT/Alert PF Plus and Bactec Peds Plus bottles showed 0% recovery (ceftriaxone with *E. coli*, *S. agalactiae*, or *S. pneumoniae*, and cefepime with *E. coli*) or partial recovery (meropenem with *E. coli* or *P. aeruginosa*). These findings are similar to those by Sullivan et al. (2013), who reported no recoveries for not only *E. coli*, *S. agalactiae*, or *S. pneumoniae* but also for *Neisseria meningitidis* or *Haemophilus influenzae* (two fastidious organisms responsible for pediatric BSI) when tested in the presence of third-generation cephalosporins (ceftriaxone or cefotaxime). Similarly, our previous study (albeit in the adult setting) showed no recovery for *E. coli* when tested in the presence of meropenem at the lowest but clinically relevant concentration in both BacT/Alert (FA Plus and FN Plus) and Bactec (Plus Aerobic/F and Plus Anaerobic/F) bottles (Menchinelli et al., 2019b). Concomitantly, Chen et al. (2019) showed that the recovery of *E. coli* exposed to meropenem midpoint and trough concentrations was more successful in BacT/Alert FN Plus bottles than in Bactec Plus Anaerobic/F bottles. However, it should be noted that the bacterial inoculum (50 to 100 CFU per bottle) used by Menchinelli et al. (2019b) was much higher than the inoculum (7 to 30 CFU per bottle) used by Chen et al. (2019), and this difference might have caused the dissimilar performance of BC bottles observed in the two studies. Overall, compared to adult counterparts (Chen et al., 2019; Menchinelli et al., 2019b), pediatric BC bottles displayed lower recovery rates, particularly at peak drug concentrations or low bacteremia levels tested. This was despite the broth-to-blood ratio in adult bottles being equivalent to that in pediatric bottles, implying that no dilution effect might have caused the above contrasting observations.

We noticed striking superiority of the BacT/Alert PF Plus bottle with ampicillin or cefepime peak concentrations and with vancomycin peak and trough concentrations. Instead, the Bactec Peds Plus bottle failed to prevent the antibiotic-mediated inhibition of growth for the organism exposed to these drugs. Conversely, Sullivan et al. (2013) noticed that the Bactec Peds Plus bottle was able to recover *Staphylococcus* spp. (MRSA and *S. epidermidis*) or *S. pneumoniae* organisms when exposed to vancomycin peak and trough concentrations. A reason for contrasting results between the studies may be the difference in the simulated level of bacteremia, namely 10 to 100 CFU/ml (Sullivan et al., 2013) or 2 to 16 CFU/ml (present study). Interestingly, we noticed that BacT/Alert PF Plus bottles also had significantly shorter TTD in both control (inoculated with antibiotic-free blood) and test (inoculated with antibiotic-containing blood) bottles. It is plausible that BacT/Alert PF Plus bottles are more enriched in some component(s) than Bactec Peds Plus bottles that may accelerate organisms’ growth. Taken together, these observations underscore the possibility for more rapid detection of BSI pathogens (Spaulding et al., 2019) using the BacT/Alert PF Plus bottles. The study further underscores the need for more caution with the timing of blood collection in patients at risk of *Staphylococcus* spp. or *S. pneumoniae* bacteremia when using the Bactec Peds Plus bottles.

The strength of this study includes the simulation of pediatric bacteremia at low or intermediate levels, mimicking the common scenario of levels in pediatric BC bottles inoculated with 2-ml blood volume. Although bacteremia is a difficult clinical entity to classify as low, intermediate (or moderate), or high, we are confident that the two experimentally made levels do actually correspond to clinical situations that may differently affect pediatric management and outcomes (Kellogg et al., 2000). We did not simulate a high bacteremia level because it seemed to represent a less common situation (Dien Bard and McElvania TeKippe, 2016). Meanwhile, our experimental design did not hamper us comparing detection (and relative TTD) in the control versus test bottles. One limitation of this study is the use of seeded banked whole blood (*i.e.*, obtained from adult donors) to simulate actual clinical samples. In this context, we acknowledged the potential inhibitory effect of adult blood because of antibodies or activation complement components, which may affect bacterial growth or may clear organisms even in the absence of antibiotics. Instead, use of clinical samples would take into account the interference by molecules involved in the bacteremia process or by antibiotics administered before sampling. Secondly, we did not determine antibiotic concentrations in test bottles at the initial and subsequent time points of inoculation to ensure consistency between the observed whole-blood concentrations and the predicted peak or trough concentrations for antibiotics used. Additionally, organisms allowed to grow in bottles containing trough antibiotic concentrations were never exposed to peak antibiotic concentrations. This would hinder assessing the post-antibiotic effect or the ratio of the area under the concentration–time curve (AUC) to the MIC, which underlies antibiotics’ exposure-to-effect relationships. Thirdly, the number of bacterial pathogens included in our study was limited, thereby excluding fastidious organisms like *N. meningitidis* or *H. influenzae*. To date, it is unknown about further BC bottles’ advances to enhance the recovery of difficult-to-grow but clinically important organisms. Fourthly, the number of replicates for each antibiotic–bacterium combination in our study could be enlarged.

In conclusion, resin-based antibiotic inactivation in BacT/Alert PF Plus bottles may be superior to in Bactec Peds Plus bottles when simulating low/intermediate bacteremia in pediatric patients on antimicrobial therapy. For both BacT/Alert Plus and Bactec Peds Plus bottles, performance was optimal with either piperacillin/tazobactam (*i.e.*, first-line antibiotic in *P. aeruginosa* bacteremia) or oxacillin (*i.e.*, first-line antibiotic in MSSA bacteremia). However, performance of both bottles was far from optimal with either ceftriaxone (*i.e.*, first-line antibiotic in *E. coli*, *S. agalactiae*, or *S. pneumoniae* bacteremia) or cefepime (*i.e.*, first-line antibiotic in *E. coli* bacteremia). Further studies will allow full appreciation of the advantages of BacT/Alert PF Plus or Bactec Peds Plus BC bottles in patients for whom empirically administered antimicrobials prior to BC sampling make detection of bacteremia particularly difficult.

## Data Availability Statement

The raw data supporting the conclusions of this article will be made available by the authors, without undue reservation.

## Author Contributions

LG, FML, and GM performed the experimental work and data analysis. GDA, TDI, and GAM helped analyze the data. MS, TS, and BP conceived the study and supervised the study conduction and the data analysis. BP wrote the paper. LG, FML, GM, and GDA helped write the paper. All authors contributed to the article and approved the submitted version.

## Funding

BioMérieux provided reagents and funding for this study, participated in the study design, and critically reviewed the manuscript before submission.

## Conflict of Interest

The authors declare that bioMérieux provided reagents and funding for this study, participated in the study design, and critically reviewed the manuscript before submission.
